# Antioxidant and antimicrobial activities and GC/MS-based phytochemical analysis of two traditional Lichen species *Trypethellium virens* and *Phaeographis dendritica*

**DOI:** 10.1186/s43141-023-00490-0

**Published:** 2023-04-04

**Authors:** Shubham Pradhan, Satyabrata Dash, Sabyasachy Parida, Bijayananda Sahoo, Biswajit Rath

**Affiliations:** Department of Biotechnology, Maharaja Sriram Chandra Bhanja Deo University, Sriram Chandra Vihar, Takatpur, Baripada-757003 Odisha India

**Keywords:** 28 s rRNA, Antimicrobial, Antioxidant, GC–MS, Lichens, PCR, Phylogeny

## Abstract

**Background:**

Lichens are complex plants living in symbiotic relationship between fungi and algae. They are used for human and animal nutrition and are used in folk medicine in many countries over a considerable period of time. In the present study, various solvent extracts of *Trypethelslium virens* and *Phaeographis dendritica* were tested for their antioxidant and antimicrobial activity.

**Results:**

The phytochemical analysis by GC/MS revealed phenolics (1.273%), terpene (0.963%), hydrocarbons (2.081%), benzofurans (2.081%), quinone (1.273%), alkanes (0.963%), and aliphatic aldehydes (0.963%) as the predominant compounds in *Trypethellium virens* SPTV02, whereas secondary alcohol (1.184%), alkaloids (1.184%), and fatty acids (4.466) were the major constituents in *Phaeographis dendritica*. The antioxidant property of methanolic extract of *T. virens* and *P. dendritica* revealed the presence of total phenolic and terpenoids. The methanolic extracts of both the lichens exhibited encouraging DPPH antiradical activity, with the IC50 of 62.4 ± 0.76 µg/ml for *T. virens* and 68.48 ± 0.45 µg/ml for *P. dendritica.* Similarly, ferric reducing power assay result exhibited higher reducing activity. Further, the lichen extracts (methanolic) indicated promising antimicrobial activities against pathogens showing MIC from 62.5 to 500 µg/ml.

**Conclusion:**

The study results concludes that both the lichens could be used as new natural source of antioxidants and antimicrobial agents which can be exploited for pharmaceutical applications.

## Background

Lichens are unique symbiotic cryptogamic flora alliances between a mycobiont, a photobiont, and/or a cyanobiont, which resemble non-flowering plants. Besides this, bacteria can repopulate lichens and form a third partner [1]. There is a wide diversity of lichen species globally that can sustain themselves in a broad array of extreme environmental conditions because of their unique impedance capacity, making them pioneer lifeforms. The intrinsic resistance of lichen is primarily due to the formation of a diverse spectrum of secondary metabolites. Lichens have about 1050 different chemical substances including aliphatic acids, depsides and depsidones, diterpenes, dibenzofurans, naphoquinones, anthraquinones, pulvinic acids, usnic acids, and xanthones [2]. These lichen metabolites are well-known for their therapeutic potential since ancient times and employed in traditional therapies for treating external burns, wounds, asthma, colds, tuberculosis, gastritis, and other ailments in humans and animals. Lichen metabolites have been characterized for their numerous biological features such as fungicidal, cytotoxic, antimicrobial, anti-inflammatory, and antioxidant [3]. These effects have been recognized and since ancient times. Recently, much attention has focused on lichens as useful sources of natural antioxidants and antimicrobial agents [4]. The synthetic antioxidants such as butylated hydroxytoluene (BHT) and butylated hydroxyanisole (BHA), previously widely used, are now found toxic. Therefore, many researchers have focused on natural antioxidants. However, antioxidant activities of lichens and their secondary metabolites are poorly known, and only few recent works provide some useful information about this subject. The biological aspect of lichen metabolites is not yet fully confirmed, although they do often have a defensive function [5] but few studies on the bioactivities of lichens have been conducted. Consequently, exploration in this area should be extended, with further efforts focused on evaluating the in vitro antioxidant and antimicrobial properties of lichen extracts. Thus, the study is carried out to detect the secondary metabolites of *Trypethellium virens* and *Phaeographis dendritica* and to determine the antioxidant, antimicrobial, and phytochemical constituents of different solvent extracts of these lichens, which are distributed predominantly on the territory of MSCB University with an intention to isolate some novel compounds for pharmaceutical applications. Thus, the studied lichen species (*Trypethellium virens* and *Phaeographis dendritica*) have been characterized which revealed the presence of phenolics, terpenes, hydrocarbons, benzofurans, quinone, alkanes, aliphatic aldehydes, secondary alcohol, alkaloids, and fatty acids. In the presence of those bioactive metabolites, both the lichens demonstrated antioxidant activity by scavenging DPPH radicals and reducing ferric ions and significant antibacterial and antifungal potency against human and plant pathogens. 

## Methods

### Study site

**Fig. 1:**
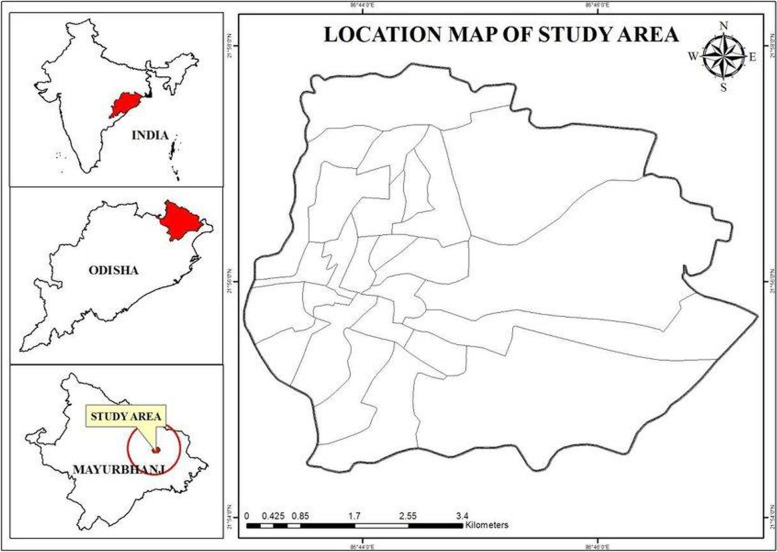
Study area: Maharaja Sriram Chandra Bhanja Deo University

Maharaja Sriram Chandra Bhanja Deo University (MSCBU) located in 21.9303° N 86.7636° E in Mayurbhanj district of Odisha State, India, closure to the Similipal Biosphere Reserve with dry deciduous vegetation types and typical eco-friendly area with significant floras. Frequent field visits were performed, and field documentation was carried out using a random sampling technique. Only distinctive samples were put together from the host and wrapped in white paper bags, packed in polythene bags, and brought to the research laboratory. The altitude was noted with a hand-held GPS (Garmin Etrex 10 × GPS), the temperature and humidity were measured by a hygrometer (HTC) seasonally, and microhabitat data was recorded for each transect (Fig. 1).

### Identification of lichens

**Fig. 2:**
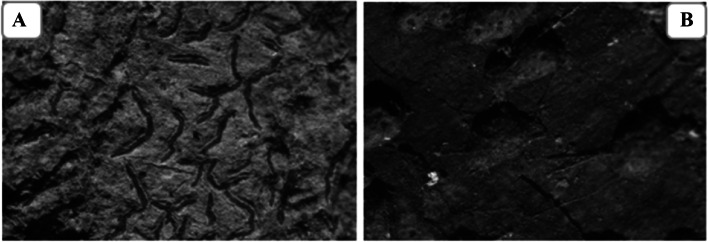
Microscopic photograph of Lichen species **A** *Phaeographis dendritica* and **B** *Trypethelium virens*

Thirteen number of lichens samples growing on tree barks were collected, separated, and identified based on their morphology using lichen identification manual [6]. Based on the predominant and diversity indexes, several lichen species were explored on campus and deposited at Department of Biotechnology (MSCB University) from which *Trypethellium virens* (Lücking, Nelsen, & Aptroot 2016) and *Phaeographis dendritica* (Ach., Müll. Arg. 1882) (Fig. 2A, B) were used for experimental purpose due to their dominance in the territory.

### DNA isolation from lichens

Lichen thallus (10 mg) was taken in a micro-vial and added three or four 2.5-mm sterile glass beads to the sample and placed in liquid nitrogen; disrupted the sample with a Mini-Beadbeater-24 for 30 s. The sample was shaken vigorously approximately 20 times vertically with KCl extraction buffer (300 µL), added chloroform (300 µL), and kept inverted. The sample was centrifuged (12,000 g) for 1 min at room temperature. The upper liquid layer was transferred to a microcentrifuge tube and added 180 µL of chilled isopropanol up to 60% total volume. Gently combined, centrifuged (12,000 g) for 1 min at room temperature, then discarded the supernatant. The resulting pellet was washed with 300 µL chilled ethanol (70%). The pellet was dried at 55^o^ C in oven for 5 min. and 100 µL of TE buffer was added. The purified DNA may be stored at 4 °C [7].

### PCR and sequencing

For the sequences generated by our research group, partial genomic DNA was isolated from mycelium (upper cortex region). Amplification of ribosomal DNA (rDNA), including the intervening internal transcribed spacer regions, 5.8S rDNA (ITS1–5.8S–ITS2), and 28 s larger sub-unit rDNA (PLN1-28 s-PLN2) was performed using the forward primers ITS1 5′-AAGTCGTAACAAGGTCTCCGTA-3′ (GC 45.5%, Tm 55.3ºC, ΔG − 39.3 kcal/mol) and the reverse primer ITS2 5′-TTAAGTTCAGCGGGTATTCCTA-3′ (GC 40.9%, Tm 53.2ºC, ΔG − 40.68 kcal/mol) for *P. dendritica* strain SPB041 and PLN1 as forward primer 5′-TATACAATAAGCGGACCTTTTT-3′ (GC 31.8% Tm 49.8ºC ΔG − 39.8 kcal/mol) and reverse primer 5′ AAAAATGGCCCACTAGTAACGC-3′ (GC 45.5% Tm 58.2ºC ΔG − 43.35 kcal/mol) for *T. virens* SPTV02 following the protocol of [8]. PCR products were visualized on a 1.5% agarose gel (120 V for 25 min) to validate the presence and size of amplicons, followed by purification via Exonuclease I and recombinant Shrimp Alkaline Phosphatase, and sequenced bidirectionally on a Capillary Electrophoresis Genetic Analyzer (ABI 3730). Forward and reverse proofreads were edited and assembled in MEGA and Sequencher v. 5 then deposited in GenBank (OP819895 and OP879718). PCR products for ITS generally range in size from 400 to 1200 nucleotides for most lichen-forming fungi that are examined.

### Preparation of the lichen extracts

Lichen samples are dried and made into powder form, from which 10 g was extracted on Soxhlet apparatus (Barosil) at 45 °C for 24 h using organic solvents (methanol, acetone, benzene, diethyl ether). After 6 to 8 heat cyclic extraction, the solvent was evaporated using a hot air oven at 42–45 °C to obtain dry extract up to 2 g each.

### DPPH radical scavenging

The radical scavenging activity of two test lichens was evaluated by DPPH (1,1-diphenyl-2-picryl-hydrazil) assay [9]. DPPH solution of 1 ml (0.1 mM) was added to 3 ml of different concentration (100–500 μg/ml) of lichen extract, and the mixture was kept for 30 min (incubation) at room temperature and the absorbance was recorded at 517 nm using UV–visible spectrophotometer (Systronics-119). The DPPH radical scavenging activity was calculated by the equation: activity (%) = [(control Abs (A_0_) – sample Abs (A_1_))/control Abs (A_0_)] × 100, where A_0_ is the absorbance of the negative control (2 ml of methanol solution of DPPH radical + 1 ml of 5% DMSO) and A_1_ is the absorbance of reaction mixture or standards, butylated hydroxytoluene (BHT) were used as standards. Ascorbic acid was used as positive control.

### Ferric-reducing activity

The ferric-reducing antioxidant power of the studied lichen species was calculated using the method of [10]. Different concentrations of two lichen extracts (100, 250, 500, 750, and 1000 μg/ml) were mixed with 2.5 ml of PO_4_ buffer (pH 6.6, 0.2 M) and potassium ferricyanide (1%) and upheld at 50 °C for 25 min. It was then blended with 10% trichloroacetic acid and centrifuged (3000 rpm) for 20 min. Afterwards, the resultant supernatant was meticulously mixed with 2.5 ml of distilled water and 0.5 ml of FeCl_3_ (0.1%), and absorbance measured at 700 nm. The assay used butylated hydroxytoluene (BHT) as a positive control.

### Determination of total phenolic contents (TPC)

The total phenolic content was determined using the Folin-Ciocalteu reagent [11]. Lichen extract (100 μl) was treated with 2 ml of sodium carbonate (2%). After a 10-min gap, 500 μl of Folin’s reagent was added, and the reaction mixture was incubated in the dark at room temperature for 20 min and the absorbance was taken at 650 nm (Spectrometer- Systronics-119). The finding was plotted as GAE/g dry extract.

### Estimation of total flavonoid content (TFC)

The total flavonoid content was determined according to the method of [12]. One milliliter of the lichen extract was mixed with 500 μl of sodium nitrite and aluminium chloride (10%, 300 μl). After 10 min, 1 ml of sodium hydroxide (1 M) was mixed and the volume was made up to 5 ml with distilled water and the mixture was incubated for 30 min and the absorbance was recited at 510 nm. The result was expressed as quercetin equivalent, i.e., μg QE/g dry extract.

### Estimation of total Tannin

The total tannin was determined by modified method of [10]. The standard (tannic acid) solution of five different concentrations (100, 200, 300, 400, and 500 µg/mL) and the lichen extract of 200 µg/mL of 0.1 mL/100 µl were taken in different test tubes. Then, 7 mL of distilled water, 0.5 mL of Folin Phenol reagent, and 1 mL of 35% sodium carbonate solution were added, and the final volume was adjusted up to 10 mL with distilled water. The mixture was shaken well, kept at room temperature for 30 min, and absorbance was taken at 725 nm on UV–Vis spectrophotometer (Jenway-119). The total tannin content of the extract was expressed as tannic acid equivalent (TAE).

### Estimation of total terpenoid

Quantitative estimation of the terpenoid content was carried out following the method of [13]. Lichen extract 1 ml (1:1 mg/ml) was mixed with chloroform (1 ml) and conc. H_2_SO_4_ (1 ml), carefully shaken, and the absorbance was taken at 538 nm. The standard curve was plotted using Linalool equivalent (mg LE/g DW) per gram dry weight of the sample taking Linalool as the standard for reference.

### Antimicrobial assays of lichens

#### Pathogens used for antimicrobial activity

Antimicrobial activity performed by using eight pathogenic microbes (four human pathogenic bacteria and fungi) is collected as microbial type culture collection (MTCC), these are *Staphylococcus aureus* (MTCC-96), *Escherichia coli* (MTCC-443), *Vibrio cholerae* (MTCC-3906), *Bacillus subtilis* (MTCC-441), *Aspergillus niger* (MTCC-1344), *Candida albicans* (MTCC-183)*, Botrytis cinerea* (MTCC-2104), and *Penicillium verrucosum* (MTCC-1558).

#### Antibacterial activity by agar well diffusion method

The anti-bacterial activity was assayed on Mueller–Hinton agar medium (MHA) by agar well diffusion [14]. Twenty milliliters of sterilized MHA was poured into sterile petri plates, and after solidification, 20 μl of pathogens (106 colony-forming unit (CFU)/ml) were swabbed on the corresponding plates. In the inoculated plates, 6-mm diameter agar wells were bored using a sterile cork borer and 50 μl of lichen extract was dissolved in dimethyl sulfoxide (DMSO) at 1 μg/μl concentration and were loaded into the respective wells and incubated at 37 °C for 24 h. After the period of incubation, the inhibition zone formed around the wells was measured and expressed in millimeter (mm) and ampicillin (0.1 μg/μl) was used as positive control and experiments were performed in triplicate.

#### Antifungal activity by agar well diffusion method

The antifungal activity was also assayed by agar well diffusion [14] on potato dextrose agar medium (PDA). The 20 ml of sterilized PDA was poured into sterile petriplates; after solidifying, 20 μl of test fungus (106 colony-forming unit (CFU)/ml) were consistently swabbed on the media plates. Agar wells of 6-mm diameter were bored using a sterile cork borer in the inoculated plates, and 50 μl of lichen extracts dissolved in DMSO (1 μg/μl) concentration were loaded into the wells and incubated at 37 °C for 24 h for 48–72 h (depends upon bacterial generation period and bacterial strain). After the incubation, the inhibitory zone shaped around the wells was measured and expressed in millimetres (mm). Clotrimazole (0.1 μg/μl) was used as a positive control, and experiments were conducted in triplicates.

#### Minimal inhibitory concentration (MIC)

This antimicrobial assay was carried out by using standard 96-well plates with Mueller Hinton broth media (MHB) for bacteria and potato dextrose broth media (PDB) for fungus to determine MIC. Lichen extracts at systematic concentrations (1000, 500, 250, 125, 62.5, 31.25, and 15.62 µg/mL) were prepared using two-fold serial dilution with MHB and a pathogen as controls. About 50 μg of the test bacterial and fungal inoculums were added at a concentration of 10^6^ CFU/mL [15]. The biofilms were analyzed after 24 h (for bacteria) and 48 h (for fungus) with a microplate reader (Biorad, iMark-11457), respectively. The value of minimal inhibition (MIC) was considered as the lowest concentration of the extract in the broth media that visible growth parameters indicate the inhibition of microbes [16].

#### Gas chromatography mass spectrometry analysis (GC–MS)

For GC–MS analysis, an extract preparation is performed using Soxhlet apparatus with methanol as solvent because methanol indicated better results as compared to other solvents (acetone, benzene, and diethyl ether). Liquid extract was collected, filtered by using Whatman’s No. 1 filter paper, afterwards dried at 45 °C for 48 h in a hot air oven, and collected as a dry extract for the characterization of biomolecules. GC–MS analyses were performed on an Elite-wax Capillary-column chromatography (60.0 m × 250 μm × 0.25 μm). The autosampler was used to inject 1.5 μl of the sample with a 10:1 split ratio. Initial temperature 60 °C for 1 min. Temperature programming to 200 °C, holding for 3 min further programmed to 10–300 °C, held for 10 min. Temperature regulation of the injection port was up to 280 °C, helium was used as a carrier gas at a flow rate of 1.0 ml/min. Solvent delay for 7 min has a transferred temperature of 160 °C, and the source temperature was 150 °C. The percentage of the extract composition was computed from the GC peak areas. A qualitative analysis that allowed for the detection was performed using PubChem, ChemSpider, Spectra Base, and the NIST standard spectral library.

### Statistical analysis

A one-way analysis of variance has been used for statistically significant correlation, followed by Duncan’s multiple range test (DMRT). Data were presented as mean ± standard deviation (SD) of three replicates. The *P* values less than 0.05 were considered significant. 

## Results

### Microhabitat

**Fig. 3:**
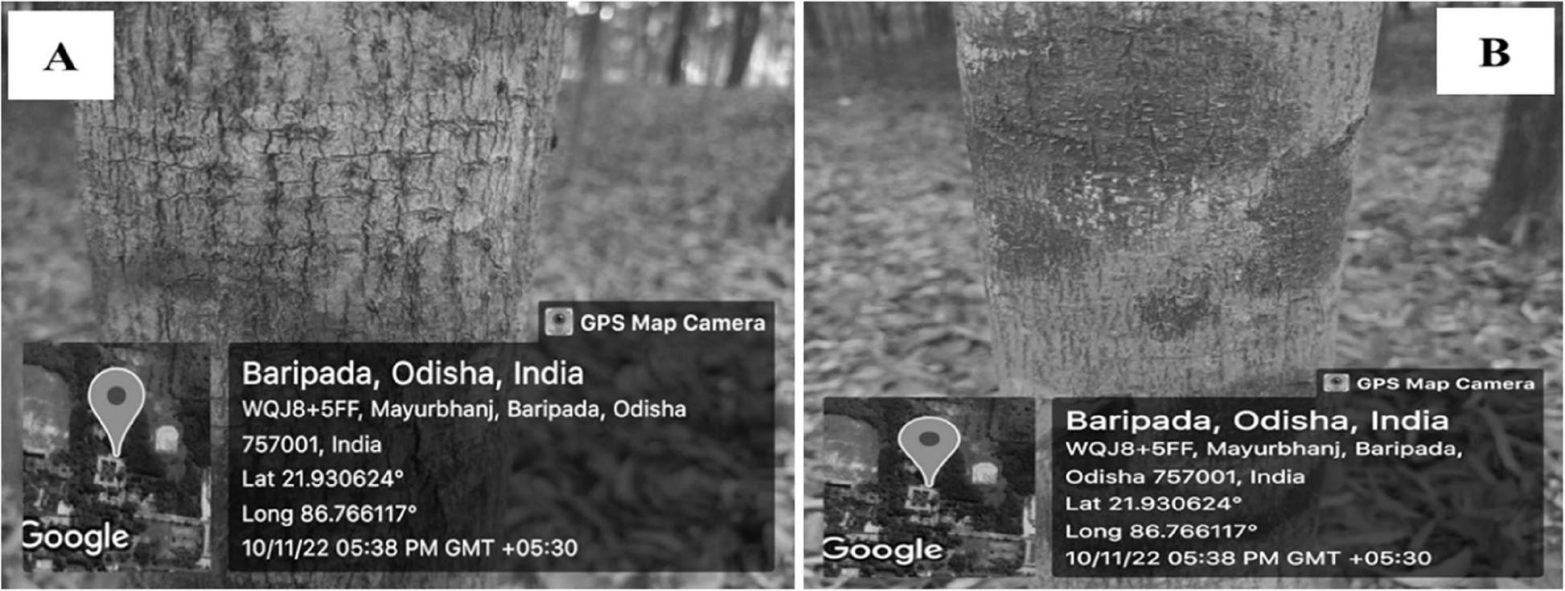
Field photography of Lichens in natural habitat **A** *Phaeographis dendritica* and **B** *Trypethellium virens*

Lichens were put together from the plant bark (Fig. 3A, B), the altitude was noted with a hand-held Garmin Etrex 10 × GPS, the relative temperature was 30 to 42 °C, and the humidity was 42 to 55% seasonally. The morphological identification was carried out and described (Tables 1 and 2).

**Table 1:** Morphological identification of Lichens

*Phaeographis dendritica*	*Trypethellium virens*
Systematic position	Systematic position
Kingdom: Fungi	Kingdom: Fungi
Division: Ascomycota	Division: Ascomycota
Class: Lecanoromycetes	Class: Dothideomycetes
Order: Ostropales	Order: Trypetheliales
Family: Graphidaceae	Family: Trypetheliaceae
Genus: *Phaeographis*	Genus: *Trypethellium*
Species: *dendritica*	Species: *virens*

**Table 2:** Morphological characteristics of lichens

Identifying characteristics	*T. virens*	*P. dendritica*
Habitat	Epiphyte (corticolous)	Epiphyte (corticolous)
Thallus type	Crustose (continuous)	Crustose (continuous)
Color	Greenish to brown	Whitish to cream-colored
Cortex	Varies in outline (45–90 mm) thick, frequently attached to bark	Variable cortex (20–85 mm) thick, frequently attached to substratum
Photobiont	*Trentepohlia* sp.	*Trentepohlia* sp.
Thalline edge	Thin, flattened, and complete	Thin and complete
Apothecia	Instead of apothecia, perithecia present	flush with the thallus or slightly elevated
Hymenium	Flattened epihymenium	Hyaline, inspersed, sparsely branching tips
Soralia, oidia, and isidia	Absent	Absent
Pseudostromata	7-mm long or width, slightly raised (Increase with thallus age)	Absent
Ostioles	Appears as black beaded in aerial view	Absent
Spores	Hyaline, septate, (38–52μ) long	Ascospores: brown, transversely septate (11–22μ)

### Molecular identification of Lichens

#### BLAST results and data validation

**Fig. 4:**
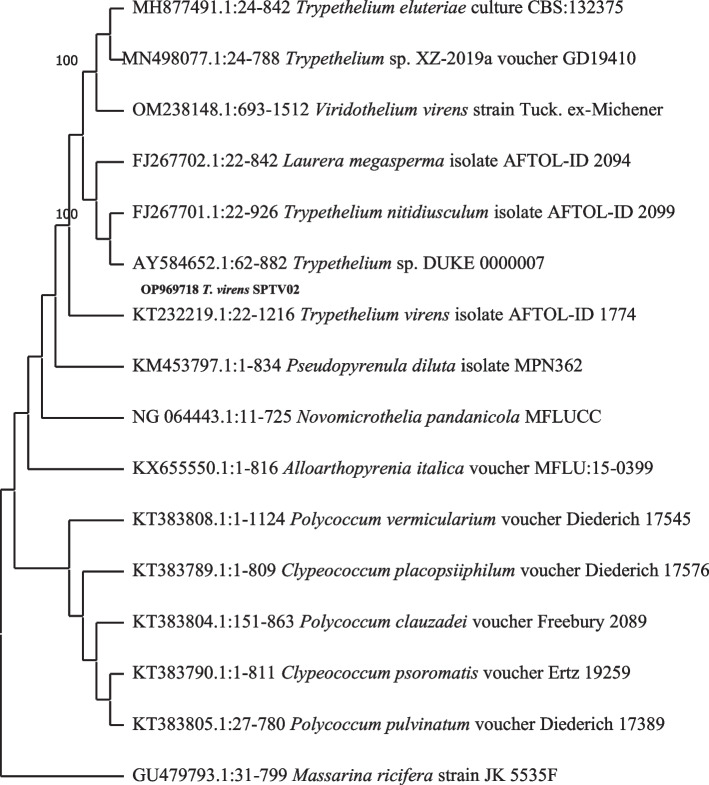
Phylogenetic relationship of *P. dendritica* across neighbor-joining method

**Fig. 5:**
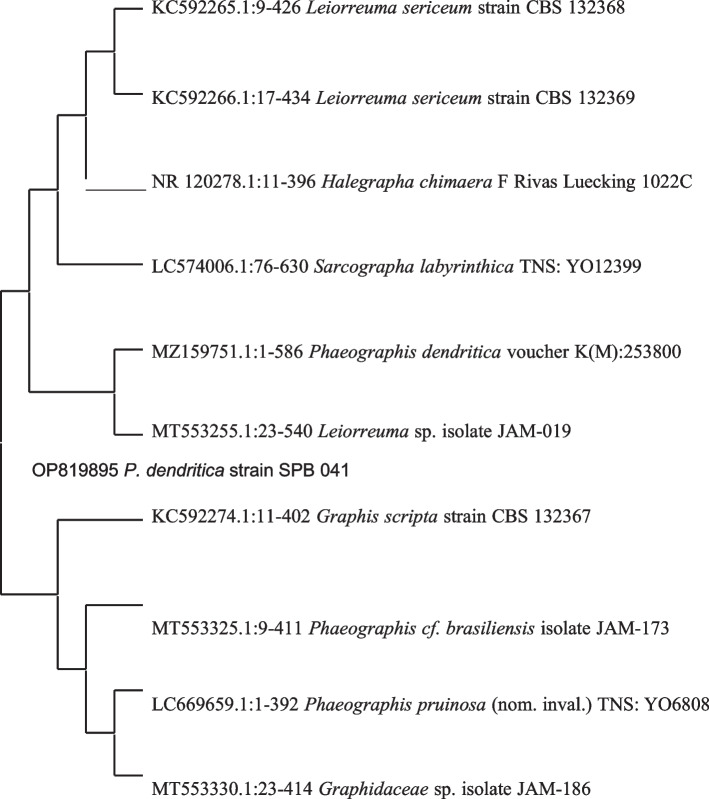
Phylogenetic relationship of *T. virens* across neighbor-joining method

A comparison of nucleotide sequences was performed using the NCBI (National Center for Biotechnology Information) database (http://www.ncbi.nlm.nih.gov/BLAST). The base pair alignment of 28S rRNA was performed using Cluster W of MEGA 11 (Molecular Evolutionary Genetic Analysis) software [17], and the aligned sequences were downloaded from the GenBank data base. A phylogenetic tree was constructed using the neighbour-joining method [18] Saitou and Nei (1987) through MEGA 11 software and tested. The bootstrap consensus tree inferred from 600 replicates is taken to signify the evolutionary history of the taxa (for both species). This analysis involved 12 nucleotide (*P. dendritica*) and 16 nucleotide (*T. virens*) sequences (Fig. 4). All ambiguous positions were removed for each sequence pair (pairwise deletion option). There was a total of 541 positions (*T. virens*) and 1238 positions (*P. dendritica)* in the final dataset (Fig. 5).

### Antioxidant activities of lichens

#### DPPH scavenging activity

**Fig. 6:**
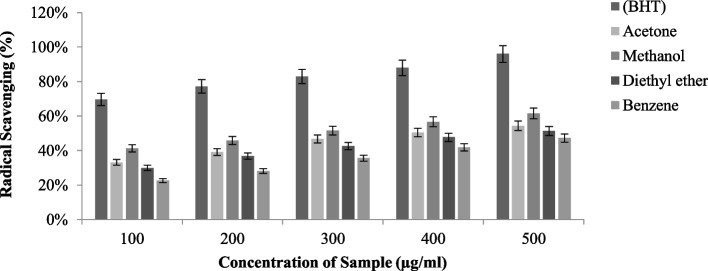
DPPH radical scavenging activity of *T. virens* in various solvent extractions

**Fig. 7:**
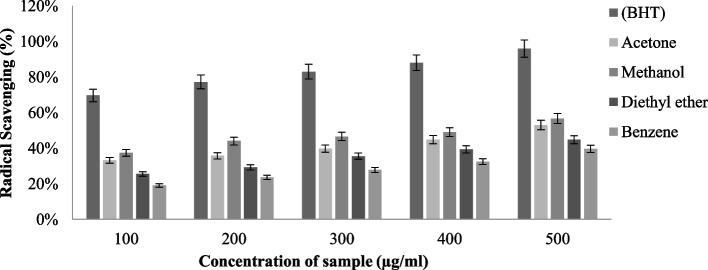
DPPH radical scavenging activity of *P. dendritica* in various solvent extractions

The DPPH radical scavenging activity was evaluated in *T. virens* (Fig. 6) and *P. dendritica* (Fig. 7) across acetone, methanol, benzene, and diethyl ether extract, which flashed different scavenging capabilities. Among other solvents, methanolic extract had maximum radical scavenging activity, having an IC_50_ (half minimal concentration) of *T. virens* (62.4 ± 0.76 µg/ml) and *P. dendritica* (68.48 ± 0.45 µg/ml), both species have strong radical scavenging potential, but *T. virens* with a minimal IC 50 value deployed relatively higher DPPH antiradical property.

#### Ferric reducing antioxidant power assay (FRAP)

**Fig. 8:**
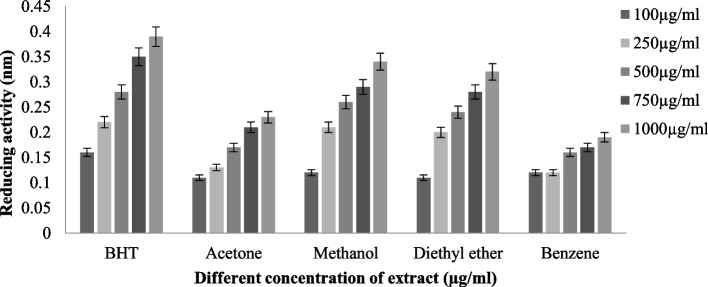
Ferric reducing activity of *T. virens*

**Fig. 9:**
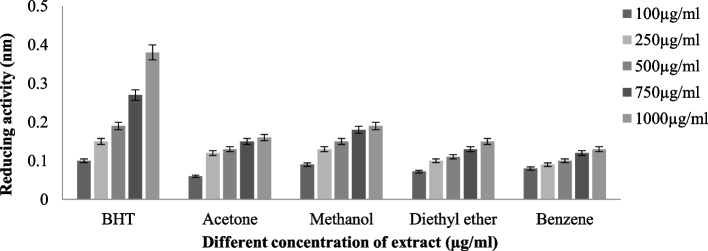
Ferric reducing activity of *P. dendritica*

The ferric ion reduction activity of tested lichen species (*T. virens* and *P. dendritica*) was prated in (Figs. 8 and 9), methalonic extracts of both species displayed higher reducing activities (*T. virens* (0.344 ± 0.011 nm) and *P. dendritica* (0.193 ± 0.003 nm)), and benzene extracts had comparably lowermost activities (*T. virens* (0.192 ± 0.012 nm) and *P. dendritica* (0.129 ± 0.03 nm)), while acetone and diethyl ether pursuit reasonably moderate reducing activity.

#### Total phenolic content (TPC)

Quantitative phenol estimation of both lichens has displayed as gallic acid calibration/equivalent (mg/g GAE of total dry extract). The higher amount of phenolics chronicled in the methalonic extract of both species, i.e., *T. virens* (0.63 ± 0.007 mg/g GAE) and *P. dendritica* (0.46 ± 0.0024 mg/g GAE). However, the extract of diethyl ether and benzene quantify moderate amount and acetone extract shows relatively lower amount of phenolics (Table 3).

**Table 3:** Quantitative phenolic estimation in *T. virens* and *P. dendritica* across organic extracts (acetone, methanol, diethyl ether, and benzene)

Total phenolics contained (mg/g) GAE		
Solvents	*T. virens*	*P. dendritica*
Acetone	0.135 ± 0.02	0.13 ± 0.005
Benzene	0.376 ± 0.017	0.22 ± 0.011
Methanol	0.63 ± 0.007	0.46 ± 0.0024
Diethyl ether	0.48 ± 0.01	0.411 ± 0.008

#### Total flavonoid content (TFC)

Estimation of total flavonoid depends on phenolics because flavonoids are classified under phenolics, but total flavonoid content was collaborated with Rutin equivalent (RE). In both tested species, methanol extraction propounded relatively better content of flavonoids in comparison to other solvents. But in a methalonic extract of *T. virens*, the maximum amount of flavonoid was recorded (0.064 ± 0.04 mg/g RE) (Table 4).

**Table 4:** Quantitative flavonoid estimation in *T. virens* and *P. dendritica* across (acetone, methanol, diethyl ether, and benzene)

Total flavonoid continent (mg/g) RE		
Solvents	*T. virens*	*P. dendritica*
Acetone	0.043 ± 0.01	0.041 ± 0.001
Benzene	0.056 ± 0.014	0.036 ± 0.4
Methanol	0.064 ± 0.04	0.041 ± 0.02
Diethyl ether	0.052 ± 0.3	0.032 ± 0.2

#### Total tannin content

The tannins are the complex phenolics, while total tannin of the test samples was calculated using the standard calibration curve of tannic acid (*y* = 0.0002*x* + 0.2029, *R*^2^ = 0.9925). Methanol and acetone extract of *T. virens* and *P. dendritica* (Table 5) propounded relatively higher content of tannin in comparison to diethyl ether and benzene extract. However, methalonic extract of *T. virens* content relatively highest (0.44 ± 0.003 mg/g TAE) tannin content.

**Table 5:** Quantitative estimations of total tannin

Total tannin content mg/g TAE		
Solvents	*T. virens*	*P. dendritica*
Acetone	0.217 ± 0.0012	0.184 ± 0.002
Methanol	0.44 ± 0.003	0.260 ± 0.007
Benzene	0.064 ± 0.004	0.03 ± 0.0006
Diethyl ether	0.134 ± 0.001	0.10 ± 0.008

#### Total terpenoid content

Total terpenoid, mentioned as isoprenoids, includes diterpenoids and triterpenoids, and the total terpenoid expressed is related to linalool equivalent (LE). Maximum terpenoid content is noted in methalonic extracts of lichens and least recorded in benzene extracts; however, methalonic extracts of *T. virens* have a higher content of terpenoid (0.252 ± 0.0012 mg/ml LE) and benzene extracts of P. *dendritica* (0.072 ± 0.003 mg/ml LE) exhibit a lower content of terpenoid content (Table 6).

**Table 6:** Quantitative estimations of total terpenoid

Total terpenoid content (mg/ml LE)		
Solvents	*T. virens*	*P. dendritica*
Acetone	0.181 ± 0.0018	0.148 ± 0.0061
Methanol	0.252 ± 0.0012	0.192 ± 0.0053
Diethyl ether	0.173 ± 0.003	0.132 ± 0.0003
Benzene	0.097 ± 0.0008	0.072 ± 0.0002

### Antimicrobial activities of lichens

#### Zone of inhibition in agar well diffusion method

**Fig. 10:**
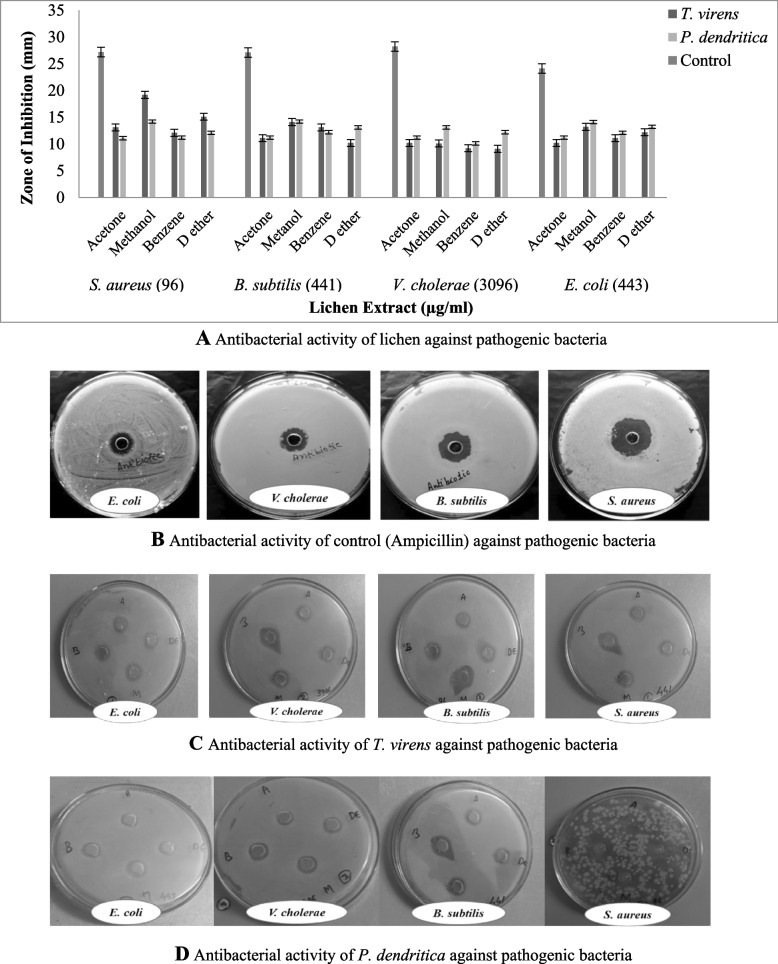
**A** Antibacterial activity of lichen against pathogenic bacteria. **B** Antibacterial activity of control (Ampicillin) against pathogenic bacteria. **C** Antibacterial activity of *T. virens* against pathogenic bacteria. **D** Antibacterial activity of *P. dendritica* against pathogenic bacteria

**Fig. 11:**
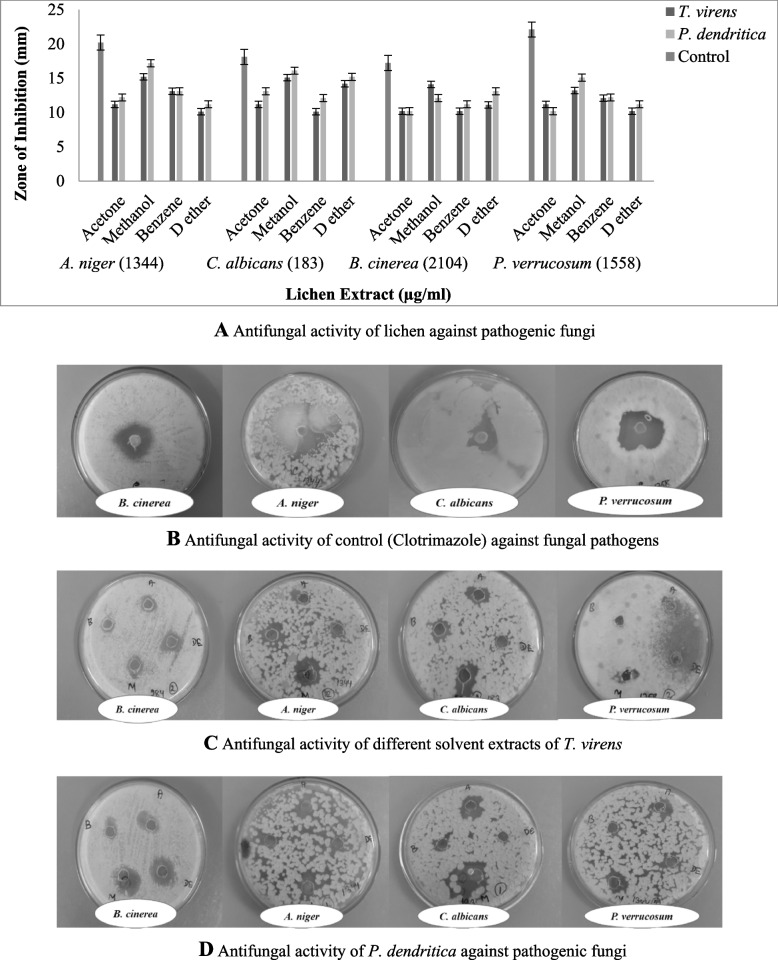
**A** Antifungal activity of lichen against pathogenic fungi. **B** Antifungal activity of control (Clotrimazole) against fungal pathogens. **C** Antifungal activity of different solvent extracts of *T. virens*. **D** Antifungal activity of different solvent extracts of *P. dendritica*

The result obtained from the antimicrobial activity lichen extracts against human pathogens (bacteria and fungus) by measuring the zone of inhibition. The zone of inhibition was depending upon solvents, concentration of the sample, and the tested pathogens. The notable antibacterial activity was viewed in methanol extract of *T. virens* (19 ± 0.2), and the zone of inhibition was also suggestively higher against gram-negative bacteria (*S. aureus*) (Fig. 10A–D). Among the organic solvents, the methalonic extract of *P. dendritica* (16 ± 0.1) showed better inhibition activity against *C. albicans*, subsequently benzene extract of *T. virens* (9 ± 0.2) showed relatively lower inhibition against *B. subtilis*. However, diethyl ether extract of *T. virens* shown moderate inhibition against *S. aureus* (15 ± 0.1) and *E. coli* (12 ± 0.2)*.* Hereafter, diethyl ether extract of *P. dendritica* displayed relatively better inhibition against *C. albicans* (15 ± 0.2); however, diethyl ether extract of *T. virens* shows minimal inhibition against *A. niger* (10 ± 0.1) and *P. verrucosum* (10 ± 0.2) (Fig. 11A–D).

#### Minimum inhibitory concentration (MIC)

The antimicrobial activity of both lichen species against the test pathogens was determined by the values of the MIC and shown in Table 7. Both selected lichens have antimicrobial potency against the pathogenic microbes. The MIC values were dependent on the solvents (which are used for extraction), their concentration, and the tested microbes. The range of MIC against the tested microorganisms was within the range of 62.5 to 500 µg/ml. *P. dendritica* liberated notable antimicrobial activity, and the MIC value was significantly lower against gram-negative bacteria than gram-positive bacteria. Among the fungal pathogens, *C. albicans* and *P. verrucosum* conferred significant MIC values.

**Table 7:** Antimicrobial activity (MIC) against pathogenic microbes across different solvent extraction

Pathogens	Minimum inhibitory concentration (µg/ml)							
	Acetone		Methanol		Benzene		Diethyl ether	
	*T. virens*	*P. dendritica*	*T. virens*	*P. dendritica*	*T. virens*	*P. dendritica*	*T. virens*	*P. dendritica*
*S. aureus*	500	250	250	125	500	250	500	500
*B. subtilis*	250	125	125	62.5	ND	500	500	250
*V. cholerae*	125	62.5	62.5	125	250	500	ND	250
*E. coli*	250	125	500	125	250	120	500	500
*A. niger*	500	500	250	250	ND	500	500	250
*C. albicans*	250	250	500	125	250	125	250	ND
*P. verrucosum*	125	125	ND	62.5	250	ND	500	500
*B. ceneria*	500	125	ND	125	ND	ND	500	125

#### GC–MS analysis and compound characterization

**Fig. 12:**
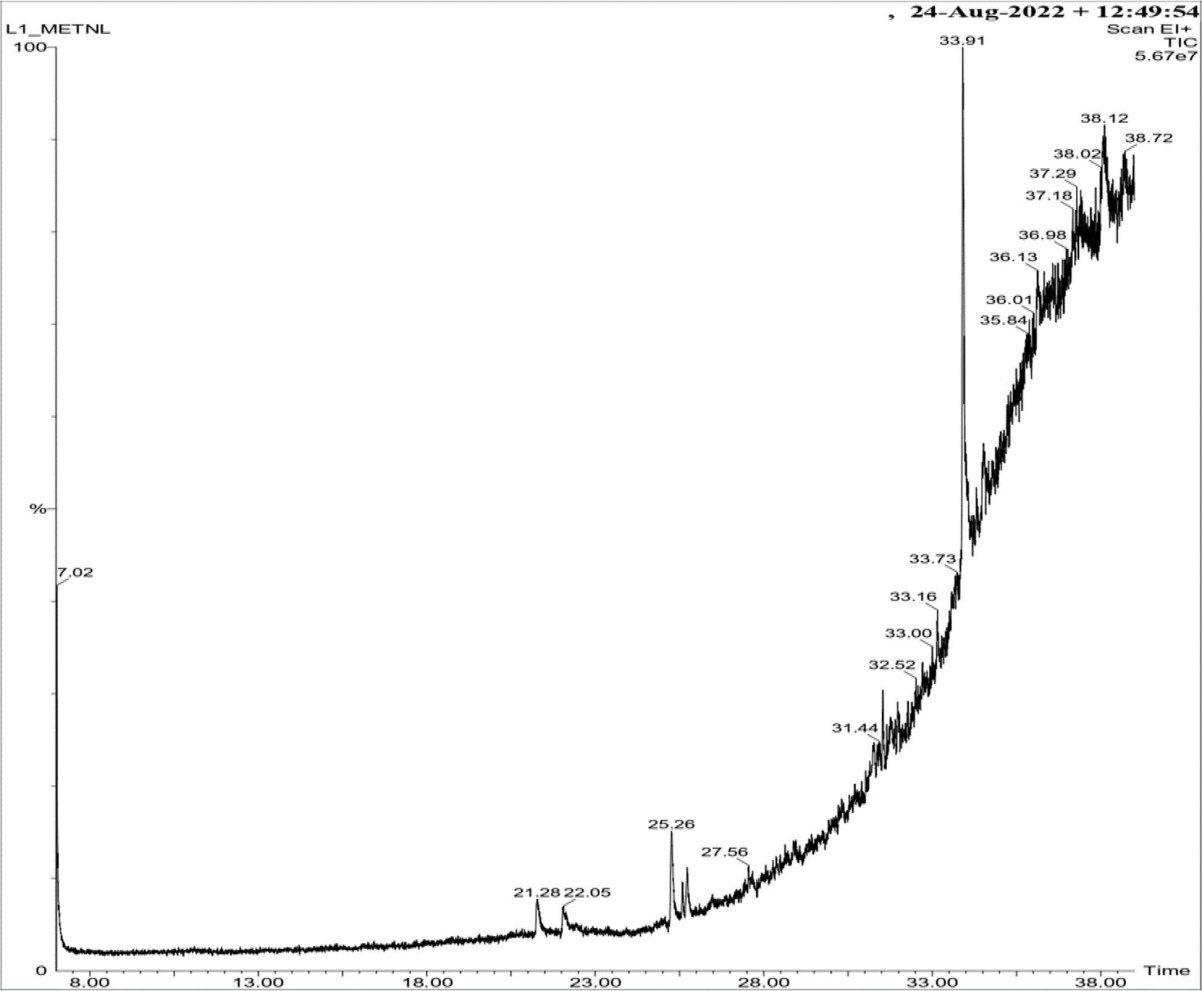
GC-MS chromatograms of methanol extract of *T. virens*

**Fig. 13:**
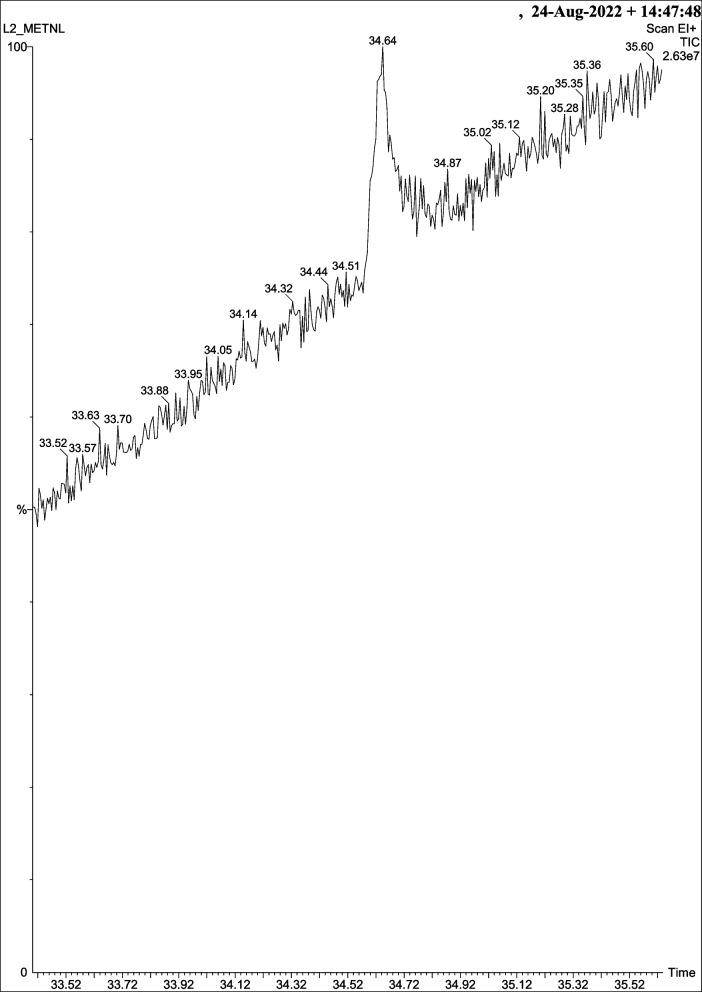
GC-MS chromatograms of methanol extract of *P. dendritica*

GC–MS screening is carried out in methalonic extracts of lichens, which detect various primary and secondary viable metabolites (Figs. 12 and 13). Some metabolites are detected through gas chromatographic techniques, and some are detected through mass spectrometry. The GC–MS screening of *T. virens* displayed recorded amounts of metabolite types such as phenolics, terpene lactones, alcohol, hydrocarbons, benzofurans, fatty acids, fluorinated aliphatic substances, organofluorine compounds, pyrans, alkanes, fatty alcohols, terpins, and aliphatic aldehydes (Table 8). However, *P. dendritica* illustrated secondary alcohol, sugar alcohol, alkaloids, and large number of fatty acids (Table 9).

**Table 8:** Compound characterization through GC–MS analysis of *T. virens*

Name of compounds	Metabolite group	Retention time	% of area	Retention index
3-METHOXY-5-PROPYLPHENOL	Phenolics	21.279	1.273	1504.3
3-Methoxy-5-methylphenol (C_8_H_10_O_2_)	phenols	21.279	1.273	1342
2,3-dimethylhydroquinone (C_8_H_10_O_2_)	Phenols	21.279	1.273	-
2,5-CYCLOHEXADIENE-1,4-DIONE	quinone	21.279	1.273	143
2-METHOXYBENZYL ALCOHOL	alcohol	21.279	1.273	2082
BICYCLO [2.2.1] HEPTAN-3-OL-5-CARBOXYLIC ACID	Terpene lactones	21.279	1.273	-
2-METHOXYBENZYL ALCOHOL	Alcohol	21.279	1.273	2082
3-Methoxy-5-pentylphenol	Phenolics	25.261	2.081	-
3-P-MENTHEN-7-AL (C_10_H_16_O)	Hydrocarbons	25.261	2.081	1196
3,4-DIMETHOXYTOLUENE (C_9_H_12_O_2_)	Hydrocarbons, Aromatic	25.261	2.081	1171
2,3-DIMETHOXYTOLUENE	Hydrocarbons, Aromatic	25.261	2.081	1806
3,6-DIMETHYL-2,3,3A,4,5,7A-HEXAHYDROBENZOFURAN (C_10_H_16_O)	Benzofurans	25.261	2.081	1178
5,9-PENTACOSADIENOIC ACID	Fatty acid	33.909	11.382	-
11,14-EICOSADIENOIC ACID	Fatty acid	33.909	11.382	-
5,9-HEXACOSADIENOIC ACID	Fatty acid	33.909	11.382	-
1,3-Dioxolane, 4-pentyl-5-propyl-2,2-bis(trifluoromethyl)-	Fluorinated aliphatic substances	38.121	0.777	-
2-TRIMETHYLSILOXY-6-HEXADECENOIC ACID	Fatty acid	38.121	0.777	-
OCTADECANOIC ACID (C_18_H_36_O_2_)	Fatty acid	38.121	0.777	2188
CYCLOHEXANEBUTANOIC ACID	Fatty acid	38.121	0.777	-
1,3-DIOXOLANE, 4-PENTYL-5-PROPYL-2,2-BIS(TRIFLUOROMETHYL)	aliphatic substance	38.121	0.777	-
1,3-DIOXOLANE, 4,5-DIPROPYL-2,2-BIS(TRIFLUOROMETHYL)-	Organofluorine compound	38.121	0.777	-
CYCLOPENTANEPROPANOIC ACID (C_8_H_14_O_2_)	carbonyl compound	38.121	0.777	2169
8-NONYNOIC ACID (C9H14O2)	fatty acid	22.054	0.624	-
UNDEC-10-YNOIC ACID (C_11_H_18_O_2_)	Fatty Acids, Unsaturated	22.054	0.624	-
PROPANOIC ACID (C_3_H_6_O_2_)	Fatty Acids, Volatile	22.054	0.624	745
4,9-DECADIENOIC ACID (C_16_H_28_O_2_)	Fatty acid	22.054	0.624	-
2-FLUORO-7-HYDROXYBICYCLO [2.2.1] HEPTANE (C_7_H_11_FO)	Fluorinated aliphatic substances	25.581	0.428	-
ACETAMIDE (C_2_H_5_NO)	Fatty Acids, Volatile	25.581	0.428	1763
CYCLOHEXENE (C_6_H_10_)	Hydrocarbons, Cyclic	25.581	0.428	674
5,7-DIHYDROXY-4-METHYLCOUMARIN (C_10_H_8_O_4_)	Pyrans	25.581	0.428	2451
BENZOIC ACID (C_7_H_6_O_2_)	Carboxylic Acid	25.731	0.761	2380
2-HYDROXY-5-METHYLISOPHTHALALDEHYDE (C_9_H_8_O_3_)	Phenols	25.731	0.761	-
CHLOROATRANORIN (C_19_H_17_ClO_8_)	Phenols	25.731	0.761	-
OLEIC ACID (C_18_H_34_O_2_)	Fatty acid	31.538	0.963	-
UNDEC-10-YNOIC ACID (C_11_H_18_O_2_)	Fatty Acids	31.538	0.963	-
11,14-EICOSADIENOIC ACID (C_20_H_36_O_2_)	Fatty Acids	31.538	0.963	-
OXIRANE, TETRADECYL- (C_16_H_32_O)	Alkanes	31.538	0.963	1708
NEOPHYTADIENE(C_20_H_38_)	terpene	31.538	0.963	-
3,7,11,15-TETRAMETHYL-2-HEXADECEN-1-OL (C_20_H_40_O)	Terpenes	31.538	0.963	-
7-Hexadecenal, (Z)- (C_16_H_30_O)	aliphatic aldehyde	31.538	0.963	2144
Undec-10-ynoic acid (C_11_H_18_O_2_)	Fatty Acids	31.538	0.963	-
9-OCTADECEN-1-OL, (Z)- (C_18_H_36_O)	Fatty Alcohols	33.159	0.731	2116
EICOSEN-1-OL, CIS-9- (C_20_H_40_O)	Long-chain fatty alcohols	33.159	0.731	2261
13-DOCOSEN-1-OL, (Z)- (C_22_H_44_O)	Fatty Alcohols	33.159	0.731	2466
PENTADECAFLUOROOCTANOIC ACID (C_8_HF_15_O_2_)	Fatty Acids	33.159	0.731	1045

**Table 9:** Characterization of metabolite screening through GC–MS analysis in *P. dendritica*

Compound	Compound type	Retention time	% of area	Retention index
3-Heptanol (C_7_H_16_O)	Secondary alcohol	13.026	1.184	1290
Xanthosine	Alkaloid	13.026	1.184	-
Pentadecanoic acid (C_15_H_30_O_2_)	Fatty acids	34.640	4.466	2821
Undecanol-4 (C_11_H_24_O)	Fatty acids	34.640	4.466	1672
N-decanoic acid (C_10_H_20_O_2_)	Fatty acids	34.640	4.466	2300
Docosanoic acid (C_22_H_44_O_2_)	Fatty acids	34.640	4.466	2567
Tetracosanoic acid (C_24_H_48_O_2_)	Fatty acids	34.640	4.466	2760
Eicosanoic acid (C_20_H_40_O_2_)	Fatty acids	34.640	4.466	2365
Mannitol (C_6_H_14_O_6_)	Sugar alcohols	34.640	4.466	-
Heptenoic acid (C_7_H_12_O_2_)	fatty acid	34.640	4.466	-
Palmitic acid (C_16_H_32_O_2_)	fatty acid	34.640	4.466	2890
Myristic acid (C_14_H_28_O_2_)	fatty acid	34.640	4.466	1765

## Discussion

The production and regulation of secondary metabolites in Lichenized fungi is complex with multivariant ecological stimulants [19] that may directly influence polyketide synthase transcription or may influence one another indirectly, initiating complex signal transduction cascades. This multifaceted system makes it difficult to separate the effects of environmental parameters, developmental stages, and other factors from one another. Stocker-Worgotter (2001) [20] proved that baeomysesic and squamatic acids were not produced by *Thamnolia* spp*.* until they were exposed to high light conditions at relatively low temperatures (15 °C). These conditions reflect the conditions in the natural habitat of *Thamnolia* spp. Larson, 1979 [21] reported that *Thamnolia* spp. typically grow in polar or alpine habitats exposed to cooler temperatures, high light conditions, and dehydrating winds, that affect water evaporation from thallus. These observations suggest that environmental parameters may trigger the production of certain compounds in some species and not the age of the Lichens. Numerous studies have shown a correlation between light levels and the production of usnic acid or other compounds within Lichen thalli [22]. The amount of atranorin in the cortex of *Parmotrema hypotropum* was shown to correlate positively with the amount of yearly light reaching the thallus. These studies concludes that chemical constituents within Lichen thallus varies more prominently because of habitat diversity, ecological conditions, and host specificity rather than age of the lichen. 

The biopotential approach has proven to be more effective in the direct measurement of phenol compounds than extraction by means of methanol in two lichen species. Furthermore, the total phenolic content ranged from 0.63 ± 0.007 of GAE/g in a methanol extract of *T. virens* to (0.46 ± 0.0024 of GAE/g) in powdered material from *P. dendritica*. Therefore, the differences are dependent on the species, the nature of the extract, and the methods of extraction; for example, Chemat et al. (2011) [23] showed that the sonicate extraction method improves the extraction of phenolic compounds, and Aoussar et al. (2016) [24] noted that the different extraction methods have an influence on antioxidant activity. The quantification of phenolics and the measurement of antioxidant capacity uses the spectroscopic approach. This showed that the total content of phenols in powdered material was high in the species studied, and it should be noted that several studies have shown that the values obtained by the quenching process are higher than those obtained by solvent extraction [25]. The total phenolic content of methanol extracts of *Flavoparmelia caperata* was reported to be 63.50 μg GAE mg by Aoussar et al. (2016) [24] and 90.83 μg by Mitrovic et al. (2011) [26], but in our findings, the content was found to be 0.311 mg/g of GAE/g. Differences in the same lichen species may be due to deviations in geographic and climatic conditions arising from the various methods of extraction [24]. Our findings corroborate other studies indicating that lichens are a rich source of phenolic compounds. Kumar et al. (2014) [27] reported, for illustration, that all the lichen extracts they studied had a significant content of alkaloids, tannins, flavonoids, and phenolic compounds. Several studies have been conducted to assess the phenolic compound and the biological properties, specifically the antioxidant activity of various lichen extracts [27, 28]. The findings of our study confirmed the abundance of phenolic compounds in lichens, which also have strong antioxidant activity in vitro against various oxidative systems such as DPPH, hydroxyl radical scavenging, and FRAP. Lichens have natural products with broad bioresource potential. Several studies have explored the biological activities of crude solvent extracts and purified compounds of lichen species in respect of their potential antioxidant, antimicrobial, antiviral, cytotoxic, insecticidal, anti-inflammatory, enzyme inhibitory, and anticancer importance [29, 30]. In our experimental work, the tested lichen extracts, using methanol, showed strong antioxidant activities and confirm the results previously obtained by Mitrovic et al. (2011) [26], who investigated the methanol extracts of the lichens *Parmelia sulcata*, *Flavoparmelia caperata*, *Evernia prunastri*, *Hypogymnia physodes*, and *Cladonia foliacea*. Furthermore, antioxidant proprieties of other lichens demonstrated their high antioxidant effect; for instance, Kumar et al. 2014 [27] showed the antioxidant capacity of 14 different lichen species by measuring various oxidative systems in vitro.

The antimicrobial activity of the methanol extracts of the examined lichens varied depending on the lichen species, extract concentration, and tested organism [31]. In this study, the methanolic extracts of *T. virens* and *P. dendritica* had the strongest antimicrobial activity among other organic extract, inhibiting the majority of the bacteria and fungi tested. It is possible that the observed differences in antimicrobial activities are due to the presence of different antimicrobial components in the extracts of different lichen species. Acknowledging the chemical components of lichens is immensely helpful because it will support in the synthesis of potentially new chemical ingredients. In recent decades, there has been a causal relationship between the total content of these compounds and the biological activities recorded in a large number of lichens. The antimicrobial properties of phenols and polyphenols are explained by the presence of phenol hydroxyl groups, whose number is correlated with their toxicity towards microorganisms. The possible mechanisms of their action include inhibition of extracellular microbial enzymes, deprivation of the substrates required for microbial growth, or direct action on microbial metabolism through inhibition of oxidative phosphorylation by sulfhydryl groups and some nonspecific interactions [32, 33]. Likewise, the presence of terpenes is observed in numerous representatives of lichens [33, 34]. According to their lipophilic nature, terpenes are observed to act by disrupting membrane functions of microbial cells, and they also cause an increase in nonspecific cell membrane permeability for the antibiotic molecule. Lichesterol have also been reported to have antibacterial properties; the correlation between membrane lipids and sensitivity to steroidal compounds indicates the mechanism by which steroids specifically associate with membrane lipids and exert their action by causing leakages from liposomes [35]. Many researchers report such phytochemical screening of various lichens [36, 37]. In the present study, we have identified and chemically characterized two crustose lichens, *T. virens* and *P. dendritica*, for the first time. The extraction of these metabolites was carried out with hot methanol. Gas chromatography (GC) identifies secondary alcohol, fatty acids, sugar alcohols, phenolics, quinone, aromatic hydrocarbons, benzofurans, carbonyl compounds, volatile fatty acids, pyrans, carboxylic acid, alkanes, aliphatic aldehydes, and long-chain fatty alcohols, whereas mass spectrometry (MS) identifies some polyphenol groups. This study reveals for the first time the different compounds of *T. virens* and *P. dendritica*, considered rare international species; moreover, it points out the status of the lichens of India as an auspicious source of bioactive molecules.

## Conclusion

The methanol, acetone, benzene, and diethyl ether extracts of two selected lichens (identified first time from MSCB University campus) displayed differential antioxidant and antimicrobial activity in vitro. Bioactive metabolites like terpene lactones, polyphenols, tannins, flavonoids, benzofurans, fluorinated aliphatic substances, organofluorine compounds, pyrans, alkanes, fatty alcohols, terpins, aliphatic aldehydes, secondary alcohols, sugar alcohols, alkaloids, and a large number of fatty acids were the major constituents of these lichens. The present investigation validates the use of lichen extracts as natural antimicrobial and antioxidant intermediaries. These lichen species can be a promising alternative to synthetic antimicrobial and antioxidant compounds and confirms that these lichens represent a significant source of phenolic compounds. Future research will concentrate on isolating different categories of phenolics and determining their biological properties in vitro and in vivo, which have a wide range of biological applications in promising drug therapies based on the lichens substances and can be of high significance in the food industry as they prevent oxidative processes, enhance quality, and nutritional value of food.

## Data Availability

The database generated and/or analyzed during in the current study is not publicly available due to privacy and confidentiality agreements as well as other restriction but is available from the corresponding author on responsible request.

## References

[1] Parrot D, Legrave N, Delmail D, Grube M, Suzuki M, Tomasi S (2016). Review–Lichen-associated bacteria as a hot spot of chemodiversity: focus on uncialamycin, a promising compound for future medicinal applications. Planta Med.

[2] Elix JA, Stocker-Wörgötter E, Nash  TH (2008). Lichen biology. ed. Nash III TH.

[3] Kosanić M, Ranković B (2019) Lichen secondary metabolites as potential antibiotic agents. Lichen secondary metabolites: bioactive properties and pharmaceutical potential 99-127.

[4] Güvenç A, Akkol EK, Süntar İ, Keleş H, Yıldız S, Çalış İ (2012). Biological activities of Pseudevernia furfuracea (L.) Zopf extracts and isolation of the active compounds. J Ethnopharmacol..

[5] Shrestha G, St Clair LL (2013). Lichens: a promising source of antibiotic and anticancer drugs. Phytochem Rev.

[6] Awasthi DD (2000). Lichenology in Indian subcontinent: a supplement to A hand book of lichens. Bishen Singh Mahendra Pal Sin.

[7] Park SY, Jang SH, Oh SO, Kim JA, Hur JS (2014). An easy rapid and cost-effective method for DNA extraction from various lichen taxa and specimens suitable for analysis of fungal and algal strains. Mycobiology.

[8] White TJ, Bruns T, Lee SJWT, Taylor J (1990). Amplification and direct sequencing of fungal ribosomal RNA genes for phylogenetics. PCR Protoc Guide Methods Appl.

[9] Kosanić M, Ranković B, Vukojević J (2011). Antioxidant properties of some lichen species. J Food Sci Technol.

[10] Oyaizu M (1986). Studies on products of browning reaction antioxidative activities of products of browning reaction prepared from glucosamine. Jpn J Nutr Diet.

[11] Taga MS, Miller EE, Pratt DE (1984). Chia seeds as a source of natural lipid antioxidants. J Am Oil Chem Soc.

[12] Zhishen J, Mengcheng T, Jianming W (1999). The determination of flavonoid contents in mulberry and their scavenging effects on superoxide radicals. Food Chem.

[13] Ghorai N, Chakraborty S, Gucchait  S, Saha SK, Biswas S (2012). Estimation of total Terpenoids concentration in plant tissues using a monoterpene. Linalool as standard reagent.

[14] Steel KJ, Barrow GI, Feltham RK (1993). Cowan and Steel’s manual for the identification of medical bacteria.

[15] Ahmad I, Khan MS, Khan RA, Khan JM (2017). Rutin inhibits mono and multi-species biofilm formation by foodborne drug resistant *Escherichia coli* and *Staphylococcus aureus*. Food Control.

[16] Srinivasan D, Nathan S, Suresh T, Perumalsamy PL (2001). Antimicrobial activity of certain Indian medicinal plants used in folkloric medicine. J Ethnopharmacol.

[17] Tamura K, Kumar S, Stecher G (2016). MEGA7: molecular evolutionary genetics analysis version 7.0 for bigger datasets. Mol Biol Evol..

[18] Saitou N, Nei M (1987). The neighbor-joining method: a new method for reconstructing phylogenetic trees. Mol Biol Evol.

[19] Fox EM, Howlett BJ (2008). Secondary metabolism: regulation and role in fungal biology. Curr Opin Microbiol.

[20] Stocker-Wörgötter E (2001). Experimental lichenology and microbiology of lichens: culture experiments, secondary chemistry of cultured mycobionts, resynthesis, and thallus morphogenesis. Bryologist.

[21] Larson DW (1979). Lichen water relations under drying conditions. New Phytol.

[22] Deduke C, Timsina B, Piercey-Normore MD (2012) Effect of environmental change on secondary metabolite production in lichen-forming fungi. International perspectives on global environmental change. InTech, 197–230.

[23] Chemat F, Khan MK (2011). Applications of ultrasound in food technology: processing preservation and extraction. Ultrason Sonochem.

[24] Aoussar N, Rhallabi RA, Mhand R, Manzali M, Bouksaim A, Douira S, Aydin K, Kinalioğlu (2016). Comparison of antioxidant activity of *Roccella*
*phycopsis* Ach. (Roccellaceae) and *Flavoparmelia*
*caperata* (L.) Hale (Parmeliaceae) lichens. Düzce Univ J Sci Technol.

[25] Kitrytė V, Šaduikis A, Venskutonis PR (2015). Assessment of antioxidant capacity of brewer’s spent grain and its supercritical carbon dioxide extract as sources of valuable dietary ingredients. J Food Eng.

[26] Mitrović T, Stamenković S, Cvetković V, Tošić S, Stanković M, Radojević I, Marković S (2011). Antioxidant, antimicrobial and antiproliferative activities of five lichen species. Int J Mol Sci.

[27] Kumar J, Dhar P, Tayade AB, Gupta D, Chaurasia OP, Upreti DK, Srivastava RB (2014). Antioxidant capacities, phenolic profile and cytotoxic effects of saxicolous lichens from trans-Himalayan cold desert of Ladakh. PLoS ONE.

[28] Kosanić M, Ranković B, Stanojković T, Rančić A, Manojlović N (2014). *Cladonia* lichens and their major metabolites as possible natural antioxidant, antimicrobial and anticancer agents. LWT-Food Sci Technol.

[29] Reddy RG, Veeraval L, Maitra S, Chollet-Krugler M, Tomasi S, Lohezic-Le Devehat F, Chakravarty S (2016). Lichen-derived compounds show potential for central nervous system therapeutics. Phytomedicine.

[30] Odabasoglu F, Aslan A, Cakir A, Suleyman H, Karagoz Y, Halici M, Bayir Y (2004). Comparison of antioxidant activity and phenolic content of three lichen species. Phytother Res.

[31] Cowan MM (1999). Plants products as antimicrobial agents. Clin Microbiol Rev.

[32] Ranković B, Kosanić M (2019) Lichens as a potential source of bioactive secondary metabolites. Lichen secondary metabolites: bioactive properties and pharmaceutical potential 1-29.

[33] Abdullah ST, Hamid H, Ali M (2007). Two new terpenes from the lichen *Parmelia perlata*. Ind J Chem.

[34] Mohammed SG (2013). Comparative study of in vitro antibacterial activity of miswak extracts and different toothpastes. Am J Agric Biol Sci.

[35] Montaño AM, Menesses R, Bravo JA, Vila JL (2016). Presence of atranorin in *Physcia*
*sorediosa*. Rev Boliv Quím.

[36] Simirgiotis MJ, Quispe C, Areche C, Sepúlveda B (2016). Phenolic compounds in Chilean Mistletoe (Quintral, *Tristerix*
*tetrandus*) analyzed by UHPLC–Q/Orbitrap/MS/MS and its antioxidant properties. Molecules.

[37] Norouzi H, Azizi A, Gholami M, Sohrabi M, Boustie J (2020). Chemotype variations among lichen ecotypes of *Umbilicaria*
*aprina* as revealed by LC-ESI-MS/MS: a survey of antioxidant phenolics. Environ Sci Pollut Res.

